# Transcriptomic Profile of Breast Tissue of Premenopausal Women Following Treatment with Progesterone Receptor Modulator: Secondary Outcomes of a Randomized Controlled Trial

**DOI:** 10.3390/ijms25147590

**Published:** 2024-07-10

**Authors:** Deborah Utjés, Nageswara Rao Boggavarapu, Mohammed Fatih Rasul, Isabelle Koberg, Alexander Zulliger, Sakthivignesh Ponandai-Srinivasan, Carolina von Grothusen, Parameswaran Grace Lalitkumar, Kiriaki Papaikonomou, Twana Alkasalias, Kristina Gemzell-Danielsson

**Affiliations:** 1 Department of Women’s and Children’s Health, Karolinska Institutet, 171 77 Stockholm, Sweden; deborah.utjes@ki.se (D.U.); nageswara.boggavarapu@ki.se (N.R.B.); mohammed.rasul@ki.se (M.F.R.); ik@koberg.net (I.K.); alexander.zulliger@ki.se (A.Z.); sakthi.ponandai.srinivasan@gmail.com (S.P.-S.); carolina.grothusen@gmail.com (C.v.G.); lalit.kumar@ki.se (P.G.L.); kiriaki.papaikonomou@ki.se (K.P.); kristina.gemzell@ki.se (K.G.-D.); 2 Department of Gynecology and Reproductive Medicine, Karolinska University Hospital, 141 57 Stockholm, Sweden; 3 Department of Pharmaceutical Basic Science, Faculty of Pharmacy, Tishk International University, Erbil 44001, Iraq; 4 General Directorate of Scientific Research Center, Salahaddin University-Erbil, Erbil 44001, Iraq; 5 WHO Collaborating Centre, Division of Gynecology and Reproduction, Karolinska University Hospital, 171 76 Stockholm, Sweden

**Keywords:** mifepristone, progesterone signaling, progesterone receptor antagonist, breast cancer

## Abstract

Progesterone receptor antagonism is gaining attention due to progesterone’s recognized role as a major mitogen in breast tissue. Limited but promising data suggest the potential efficacy of antiprogestins in breast cancer prevention. The present study presents secondary outcomes from a randomized controlled trial and examines changes in breast mRNA expression following mifepristone treatment in healthy premenopausal women. We analyzed 32 paired breast biopsies from 16 women at baseline and after two months of mifepristone treatment. In total, 27 differentially expressed genes were identified, with enriched biological functions related to extracellular matrix remodeling. Notably, the altered gene signature induced by mifepristone in vivo was rather similar to the in vitro signature. Furthermore, this gene expression signature was linked to breast carcinogenesis and notably linked with progesterone receptor expression status in breast cancer, as validated in The Cancer Genome Atlas dataset using the R2 platform. The present study is the first to explore the breast transcriptome following mifepristone treatment in normal breast tissue in vivo, enhancing the understanding of progesterone receptor antagonism and its potential protective effect against breast cancer.

## 1. Introduction

The majority of breast cancers are related to reproductive factors [1], implicating endogenous cyclic hormonal exposure affecting breast tumorigenesis. Progesterone has emerged as a major mitogen since the proliferation of breast epithelial cells occurs during the progesterone-dominated luteal phase [2,3,4]. Among different epithelial cell subtypes, the luminal progenitors serve as breast cancer precursor cells [5]. Progesterone acts primarily via a paracrine mechanism to stimulate the proliferation of the dominating progesterone receptor (PR)-negative breast cells [2], largely mediated by the downstream mediators RANK-L and WNT4 [3,6,7]. High mammographic density (HMD) is another important risk factor for breast cancer [8,9]. Therefore, the architecture and crosstalk between the stroma, including the extracellular matrix (ECM) and epithelial cells, in the context of progesterone exposure may play a vital role in the breast cancer initiation process.

The mitogenic action of progesterone can be counteracted by PR modulators. There are promising but limited data suggesting the potential efficacy of antiprogesterone in breast cancer prevention [10,11]. In premenopausal normal breast tissue, mifepristone treatment has been shown to decrease epithelial cell proliferation, reflected by significantly reduced Ki-67 expression when values at the end of treatment were compared with baseline (*p* = 0.012) [10]. Moreover, this antiproliferative impact has been confirmed when a significantly reduced WID-Breast29 epigenetic index, reflective of the mitotic age, was observed in breast tissue from healthy and *BRCA* mutation carriers (*p* = 0.003). Also, the luminal progenitor cell proportion was significantly decreased following mifepristone treatment in both groups (*p* = 0.008) [11].

Nevertheless, numerous studies have investigated the use of a low continuous dose of antiprogesterone in benign gynecological conditions and breast cancer treatment [12]. We hypothesized that antagonizing progesterone signaling may protect against breast carcinogenesis, warranting further clinical and molecular investigations. As a secondary outcome of our randomized controlled trial (RCT) [13], we studied the effects of the PR-antagonist mifepristone on normal breast tissue following two months of treatment in healthy premenopausal women. Transcriptomic profiling can improve our understanding of progesterone and antiprogesterone action in the breast and the potential breast protective effects of a PR modulator.

## 2. Results

### 2.1. Modulation of Gene Expression by Mifepristone Enriches ECM Signaling Pathways in Normal Breast Tissue

We compared the gene expression profile in normal breast tissue before and after mifepristone treatment. A false discovery rate (FDR) of ≤0.05 and a fold change (FC) of ≥2 or ≤−2 were considered statistically significant. We identified 27 differentially expressed genes (DEGs), of which 19 genes were upregulated and 8 genes were downregulated (Table 1).

We grouped the 27 DEGs and named them Gene signature Enriched to Mifepristone’s action on normal Breast (GEM-B). GEM-B represents a set of genes responsive to mifepristone in normal breast tissue. A volcano plot displaying GEM-B among overall gene expression is presented in Figure S2A. To technically validate RNA-seq data at the individual gene level, we employed real-time (RT)-PCR on the same RNA extracted samples for several genes from the GEM-B (Figure S2B). In line with the transcriptome analysis, the mifepristone-treated samples exhibited a significant upregulation of the six validated genes by RT-PCR.

To explore the biological context of the DEGs, gene functional enrichment analysis was performed using the g:Profiler database. The results of the top five terms in each of the three gene ontology (GO) categories (BP: biological process, CC: cellular component, MF: molecular function) annotated in the database are presented for the upregulated genes in Table 2.

The Reactome pathway analysis demonstrated 54 significantly enriched pathways of the upregulated DEGs. The top ten of those pathways were mainly associated with ECM organization (Table S2).

The same analyses with the downregulated DEGs revealed one significantly enriched term in the GO functional annotation (ontology: MF), namely ‘active borate transmembrane transporter activity’ with the involvement of solely gene *SLC4A1*. In the Reactome pathway analysis, there were two genes involved separately in six pathways: gene *IL1B* (involved in ‘CLEC7A/inflammasome pathway’, ‘Interleukin-1 processing’, ‘cell recruitment’ and ‘purinergic signaling in leishmaniasis infection’) and gene *LAMA1* (involved in ‘laminin interactions’ and ‘MET activates PTK2 signaling’).

The analysis was validated via a third database, Metascape-designet database. The data set enrichment from designet pathway indicated similarly the enrichment of ECM-related pathways (Figure S3).

### 2.2. The In Vivo Effect of Mifepristone Is Partially Comparable to Its In Vitro Effect on Normal Breast Tissue

To further validate the in vivo changes in the transcriptomic signature induced by mifepristone treatment, we isolated primary breast epithelial cells and exposed them to varying concentrations of mifepristone (0, 5, 50, and 100 μM) during two different treatment periods (one and three days). Different treatments were chosen in order to study the dose–response effect as well as the influence of treatment duration.

**Figure 1:**
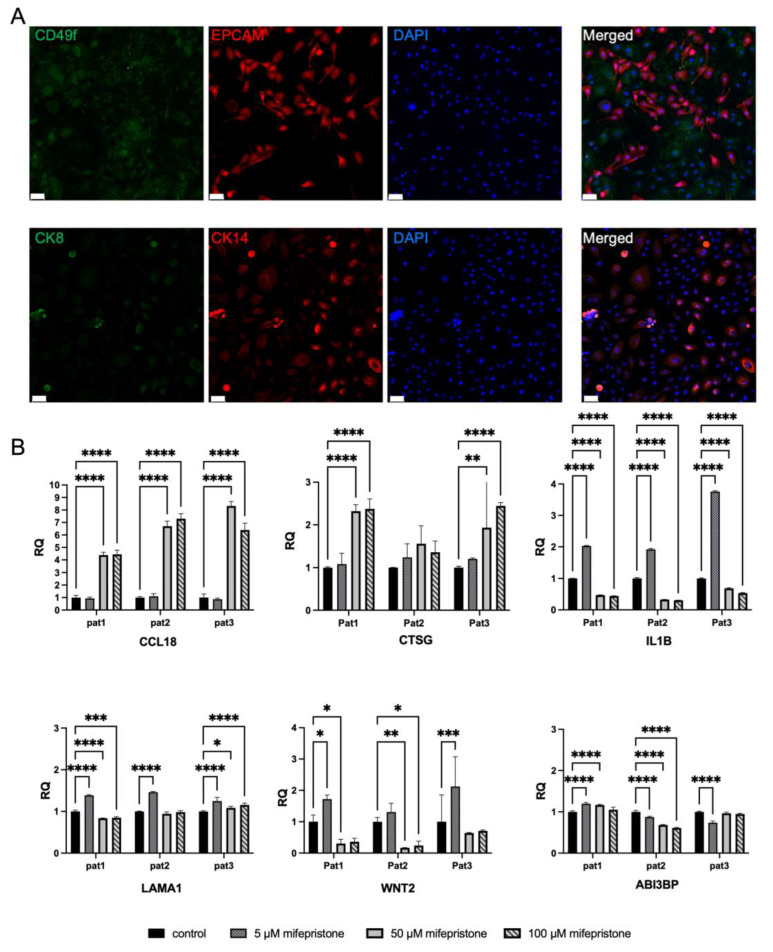
(**A**) Characterization of normal primary breast cells (basal, luminal progenitor and mature cells). The upper panel displays immunofluorescent images depicting breast cells stained with CD49f (green) and EPCAM (red), while the lower panel showcases cells stained with CK14 (red) and CK8 (green). Scale bars indicate 50 µm. DAPI was used to detect the nuclei. (**B**) Assessment of relative gene expression in normal primary breast cells. Real-time PCR analysis was conducted on selected genes (*CCL18*, *CTSG*, *ABI3BP*, *WNT2*, *IL1B*, *LAMA1*) to evaluate their expression levels in normal primary breast cells treated with varying concentrations of mifepristone (control, 5 μM, 50 μM, 100 μM) for a three-day duration. Statistical significance is denoted as follows: * *p* < 0.05; ** *p* < 0.01; *** *p* < 0.001; **** *p* < 0.0001. A scale bar indicating 50 µm is included in the figure for size reference.

First, we characterized the primary isolated cells and assessed the enrichment of distinct epithelial cell subtypes, including luminal progenitor, mature luminal, basal, and other stromal cells. The expression of four protein markers (EPCAM, CD49f, CK8, and CK14) was examined [14]. Notably, we identified diverse expression patterns; some cells exhibited positivity for CD49f or CK14, indicating a basal phenotype. Mature luminal cells expressed EPCAM or CK8. In contrast, other cells were positive for both CD49f and EPCAM, suggesting a luminal progenitor phenotype (Figure 1A). These findings underscore the significance of the heterogeneity within the isolated cells, emphasizing the need to capture the holistic impact of the drug during in vitro treatment.

Based on our analysis of RNA-seq data from the in vivo clinical trial, six candidate genes were selected among the upregulated and downregulated ones (*CCL18*, *CTSG*, *ABI3BP*, *LAMA1*, *IL1B*, *WNT2*) (Figure 1B and Figure S4). These six genes are described in the literature, most of them in relation to breast carcinogenesis and were therefore selected for this purpose [15,16,17,18]. Consistent with our findings from the RNA-seq data, the expression levels of *CCL18* and *CTSG* were upregulated in the in vitro experiment after longer treatment with higher concentrations, and the same was observed following shorter treatment (except for one patient where *CTSG* expression showed a non-significantly upregulated trend and remained unaffected, respectively). *WNT2* demonstrated upregulation in all patients when treated with low doses over three days. Conversely, it exhibited downregulation at higher concentrations during both treatment periods. Notably, *ABI3BP* demonstrated considerable inter-patient variability; it was both up- and downregulated following different concentrations seen in both treatment periods. *LAMA1* was not aligned with its downregulated pattern seen in the RCT cohort; it was mainly upregulated following treatment in vitro. However, the second-most-downregulated DEG, *IL1B*, was significantly reduced with higher concentrations of mifepristone following longer treatment, and the same was seen for two patients following shorter treatment. Interestingly, *IL1B* showed a significant upregulation with low doses of mifepristone as compared to the untreated cells.

### 2.3. GEM-B Is Linked with Breast Carcinogenesis

**Figure 2:**
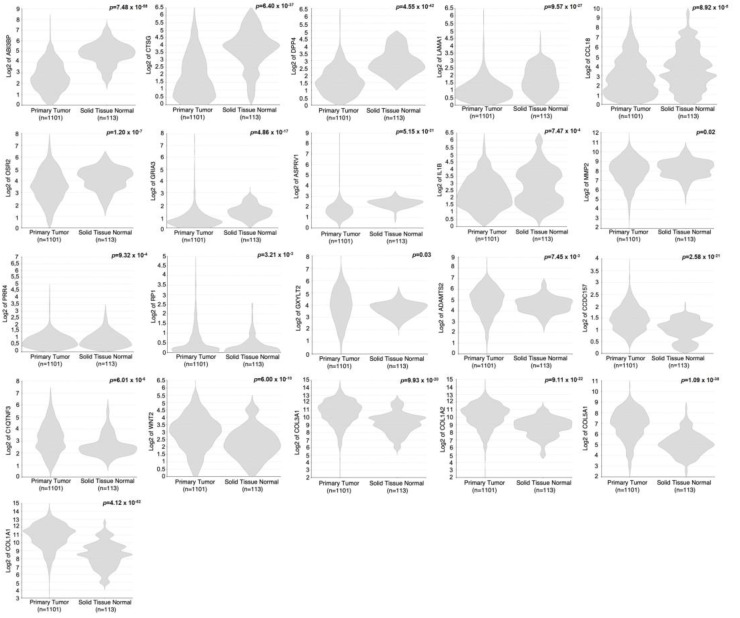
GEM-B signature enrichment in breast cancer. Utilizing the R2 platform and the TCGA breast cancer dataset, the expression patterns of each gene within the GEM-B signature were examined. Comparative analysis involved RNA-seq data between two the groups of primary breast cancer tissue (*n* = 1101) and adjacent normal breast tissue (*n* = 113). Up- and downregulated DEGs are compiled and presented gradually by enrichment status in each condition.

Given the recognized protooncogenic effect of progesterone in breast carcinogenesis, we aimed to explore the enrichment of the GEM-B signature within breast cancer samples. To achieve this objective, we systematically examined the expression patterns of our GEM-B signature in the breast cancer dataset from The Cancer Genome Atlas (TCGA) using the R2 platform. The signature was examined by comparing the RNA-seq data of primary breast cancer tissue (*n* = 1101) to adjacent normal breast tissue (*n* = 113). The results revealed a notable enrichment of GEM-B in the TCGA data cohort when comparing cancerous to normal tissue. Specifically, 21 out of the 27 signature genes exhibited significant and differential expression when comparing breast cancer to normal breast tissue. However, this enrichment displayed a dichotomy between cancerous and normal tissue. Among the downregulated DEGs, four genes (*LAMA1*, *ASPRV1*, *IL1B*, *PRR4*) displayed reduced expression, while two genes (*CCDC157*, *RP1*) showcased elevated expression in tumor compared to normal tissue, respectively. Other downregulated DEGs did not display notable differences. In terms of upregulated DEGs, seven genes (*ABI3BP*, *CTSG*, *DPP4*, *CCL18*, *OSR2*, *GRIA3*, *MMP2*) demonstrated reduced expression in tumor compared to normal tissue. In contrast, eight genes (*COL1A1*, *COL5A1*, *COL1A2*, *COL3A1*, *WNT2*, *C1QTNF3*, *ADAMTS2*, *GXYLT2*) exhibited elevated expression in tumor compared to normal tissue. The remaining upregulated DEGs did not exhibit substantial differences, as shown in Figure 2. Our findings underscore a linkage between GEM-B, comprising approximately 77.7% of the signature (21 out of 27 genes), and breast carcinogenesis.

### 2.4. GEM-B Is Substantially Linked to PR Expression Status in Breast Cancer

Having investigated the impact of mifepristone on healthy women, we aimed to explore the potential relevance of the transcriptomic changes resulting from mifepristone treatment in our cohort on the PR status of the breast cancer cohort. The TCGA breast cancer dataset facilitated the stratification of the PR status across the entire cohort, with 777 patients classified as PR-positive and 337 patients as PR-negative. We conducted an in-depth analysis of the GEM-B gene list, focusing on delineating the PR status distinctions within the breast cancer datasets.

**Figure 3:**
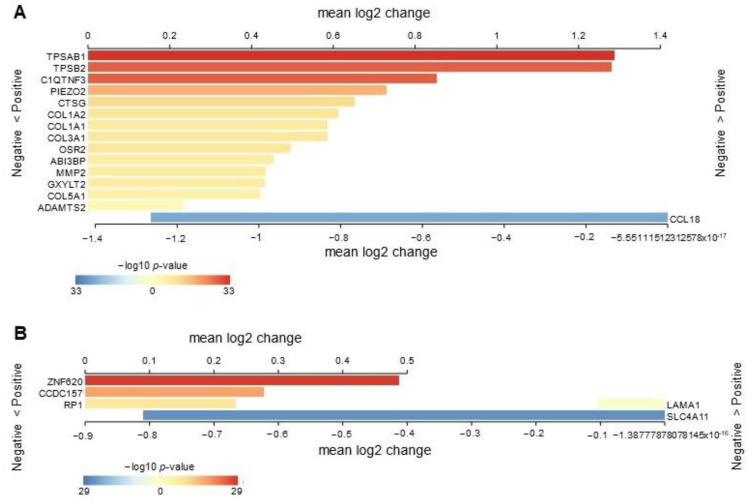
Mifepristone-driven transcriptomic changes in the context of PR status in breast cancer. Employing the R2 platform and the TCGA breast cancer dataset, seventeen genes within the GEM-B signature show significant enrichment in PR-positive cohort, whereas three genes were enriched in the PR-negative cohort. The results are presented as a heatmap illustrating the mean log2 values for each gene, with *p*-values indicated on a log10 scale. (**A**) Shows the upregulated DEGs. (**B**) Shows the downregulated DEGs. The analysis involved RNA-seq data from 1114 breast cancer tissues, comparing PR-positive cases (*n* = 777) to PR-negative cases (*n* = 337).

Out of the 27 genes within the GEM-B signature, 20 exhibited significant enrichment when comparing the PR status categories. Notably, the majority of the enriched genes demonstrated a robust correlation with PR expression. Specifically, 17 out of the 20 enriched genes (upregulated DEGs: *TPSAB1*, *TPSB2*, *C1QTNF3*, *PIEZO2*, *CTSG*, *COL1A2*, *COL1A1*, *COL3A1*, *OSR2*, *ABI3BP*, *MMP2*, *GXYLT2*, *COL5A1*, *ADAMTS2*; downregulated DEGs: *ZNF620*, *CCDC157*, *RP1*) exhibited higher expression levels in PR-positive cancer tissue compared to PR-negative counterparts, while only 3 genes (upregulated DEG: *CCL18*; downregulated DEGs: *SLC4A11*, *LAMA1*) displayed heightened enrichment in PR-negative tissue (Figure 3A,B).

## 3. Discussion

In the present study, we performed transcriptomic profiling and subsequent bioinformatics analyses to explore gene expression response associated with antagonizing progesterone in the breast tissue of healthy premenopausal women. The study focuses on the role of endogenous progesterone, enlightening the impact of mifepristone in driving the gene expression patterns and ECM signaling pathways. The findings indicate a substantial linkage of our enriched signature with the PR expression status in breast cancer.

Menstrual cycle-driven intermittent progesterone exposure and mammary gland regression have been emphasized as important causes of tumorigenesis, as opposed to gradual and continuous elevations during pregnancy or anovulation including lactational amenorrhea [1,2]. RNA-seq from normal breast tissue in the Komen bank allies with progesterone’s mitogenic role. About 87% of the upregulated genes in the luteal phase emphasize the paracrine action of RANKL, WNT4, and epiregulin as well as the enriched functions of DNA replication, mitosis, and DNA repair [19].

To address rising breast cancer incidence [8], exploring novel preventive agents is crucial. Mifepristone, a widely studied PR modulator in various benign gynecological conditions and breast cancer inhibition [12], may also hold potential in breast cancer prevention. In a rodent model, mifepristone had a reverse effect on murine mammary stem cell expansion and progesterone’s paracrine effectors [20], although caution is needed in translating animal-based results to human in vivo conditions. Only two placebo-controlled trials have assessed the effect of mifepristone in normal premenopausal human breast tissue in vivo. Exposure to mifepristone for two or three months significantly reduced Ki-67 expression in breast tissue [10], suggesting the inhibition of breast epithelial cell proliferation, and decreased mitotic age surrogate markers and luminal progenitor cell fractions in all analyzed healthy controls [11].

Our findings highlight the enrichment of several pathways following mifepristone treatment that directly regulate and drive extracellular structure organization and function. ECM displays a pivotal role in tissue homeostasis; consequently, the dysregulation and destruction of ECM dynamics can lead to tumorigeneses and cancer development [21,22]. During the menstrual cycle, it undergoes hormonal regulation, affecting cell signaling and cancer pathways in the mammary gland and the surrounding microenvironment [2]. Clinically, HMD is positively associated with collagen, ECM density, and the epithelial and stromal compartments but is negatively associated with fat tissue [8,9]. One of the main structural ECM proteins is collagen, which represents a key factor that provides tensile strength to the ECM [22], and in the present study, different collagens (*COL1A1*, *COL1A2*, *COL3A1*, *COL5A1*) were significantly enriched upon mifepristone treatment. Moreover, both collagen degradation and formation emerged as enriched pathways in our material, reflecting an increased remodeling of the ECM compared to baseline. These findings may reflect an ongoing adaptation to mifepristone, and a longer treatment protocol might have revealed the eventual direction in which the equilibrium would shift. Nevertheless, it seems that the regulation of ECM plays a central role in progesterone action and PR antagonism in the breast.

A fundamental approach for assessing progesterone signaling involves the assessment of PR expression and the subsequent downstream actions within the signaling pathway. The notable enrichment of 20 genes within the GEM-B signature in PR-positive breast cancer tissue underscores the substantial involvement of progesterone in the development of breast cancer. Furthermore, our findings suggest that blocking progesterone signaling with mifepristone may play a significant role in preventing the initiation of breast cancer. This prompts further avenues for in-depth mechanistic studies, shedding light on mifepristone’s potential as both a preventive and therapeutic strategy for breast cancer.

To the best of our knowledge, the present study is the first to explore the changes in the transcriptomic landscape and biological functions following progesterone antagonism with mifepristone treatment in healthy breast tissue in vivo. The sample population originates from a double-blind RCT [13], limiting the bias in the results, and individual paired samples were used, thus reducing the inter-individual variability. Next-generation sequencing (NGS) was used to identify DEGs, and the results were validated with RT-PCR, confirming the expression pattern for all six randomly chosen genes; this reinforced that data derived from RNA-seq technology are of a robust nature and could be applied for further analyses. However, long-term effects after treatment discontinuation were not elucidated due to a lack of follow-up data. Based on indications of ECM remodeling following mifepristone treatment, measurements of breast stiffness and density through mammographies could provide further insights. Even though breast cancer seems to arise predominantly from epithelial cells [2], the stroma of the mammary gland, comprised mainly of the ECM, emerged in our study as playing a key role. This is in line with a plethora of investigations on breast cancer, even suggesting ECM remodeling as a potential therapeutic target [22].

Moreover, mifepristone’s induced changes in the mRNA level of the selected genes from the in vitro validation approach did not match their expression level in ex vivo breast biopsies. The 2D cell culture has completely different environment from the in vivo condition, where with 2D cultures are derived from enzymatically degraded normal breast tissue to obtain single cells. Eventually, this disrupts the tissue architecture supported by the interaction and crosslinking of ECM components. Hence, the genes coding for ECM pathways are not regulated in the same manner. Additionally, the treatment period, metabolism, and dosage are challenging to replicate in an in vitro setup. This validation highlights the challenges of mimicking the in vivo environment in vitro.

### Limitations and Future Directions

Despite our RCT representing a unique trial [13], given the restrictions on mifepristone applications in several European and Western countries, the relatively small dataset used in our study presents a limitation. To overcome this, future studies should leverage larger datasets to enhance the reliability and generalizability of the results. Integrating external datasets and implanting self-supervised learning tools such as generative adversarial networks [23,24], where applicable, can significantly augment statistical power and provide a more comprehensive understanding of the underlying biological processes. Additionally, due to the small sample size, we acknowledge the limitation of not validating the DEGs using an orthogonal method. A future approach should involve employing additional validation techniques such as Western blotting and utilizing a bigger biopsy, if possible, to comprehensively verify the findings. This approach would provide a more robust confirmation of the DEGs and enriched pathways identified in our study.

## 4. Methods and Materials 

This study reports the secondary outcome of a prospective, double-blind, placebo-controlled RCT. The primary objective was to study the impact of pretreatment with a continuous low dose of mifepristone on menstrual bleeding patterns in women opting for a levonorgestrel-releasing intrauterine device for contraception, and the results are published by our group elsewhere [13]. The secondary aim presented here was to investigate the effect of mifepristone treatment on breast tissue. The study was conducted at Karolinska University Hospital, Stockholm, Sweden, from 2009 until 2015 and was approved by the Swedish Medical Products Agency (EudraCT number 2009-009014-40). The study protocol was designed according to the recommendations in the CONSORT statement and was approved by the ethical committee at Karolinska Institutet (Dnr: 2009/144-31/4) prior to recruitment. The trial was registered at clinicaltrials.gov (NCT01931657).

### 4.1. Subjects

Eligible study subjects were healthy premenopausal women aged 18–43 years with regular menstrual cycles lasting 25–35 days and with no contraindications to any of the study treatments. All exclusion criteria are presented in the original study, including the use of any hormonal or intrauterine contraception, pregnancy, or breastfeeding two months before the study, or a history of breast cancer or other malignancies. The trial flowchart explains the details of the enrolled subjects in the current cohort (Figure S1), and the baseline characteristics of the women contributing to paired breast biopsies analyses are presented in Table S1.

### 4.2. Treatment

Study subjects were randomized into two treatment groups [13]. One group was treated with 50 mg mifepristone (one-quarter of 200 mg Mifegyne^®^, Exelgyn, Paris, France) every other day for two months (56 days), starting on the first day of the menstrual cycle. The comparator group received visually indistinguishable B-vitamin tablets (TrioBe^®^ Recip), which were also divided into four parts. For the purpose of the present study, only paired breast samples from the mifepristone-treated group were analyzed.

### 4.3. Biopsy Collection

Core needle breast aspiration biopsies were collected at baseline and at the end of the treatment, under ultrasound guidance from the upper-outer quadrant of one breast using a 14-gauge needle with an outer diameter of 2.2 mm. The collected breast tissue was divided into two parts, snap-frozen, and stored at −180 °C until further processing.

In order to perform a functional validation with an in vitro experiment, we used breast tissue samples from three additional healthy and premenopausal women undergoing mammoplasty procedures. This collection was performed according to a separate study, approved by the ethical committee at Karolinska Institutet (Dnr: 2021-04144) prior to recruitment.

### 4.4. RNA Extraction

RNA extraction was performed on 16 paired samples (i.e., 32 samples) using the PurelinkTM RNA Micro kit in conjunction with TRIzol reagent (Life Technologies, Carlsbad, CA 92008, USA). For in vitro studies, the RNA extraction from primary breast cells was performed using Quick-DNA/RNATM Microprep Plus; Zymoresearch. RNA quantification was conducted using the Qubit RNA High Sensitivity Assay Kit (Invitrogen/Thermo Fischer, Waltham, MA, USA).

### 4.5. cDNA Library Construction and Sequencing

Complementary DNA (cDNA) libraries for NGS were constructed from RNA for the 16 paired samples before and after mifepristone treatment, using the well-established Smart-seq2 protocol [25]. Moreover, 1 ng RNA from each sample was taken as starting material for NGS library construction based on the Qubit quantification. Tagmentation of the cDNA was performed using a Nextera XT Kit (Illumina) followed by the addition of adapters and index primers, as per the Nextera XT kit recommendations. The resulting DNA libraries, after Nextera reactions, were purified using AMPure XP beads (Beckman coulter, Brea, CA, USA) in 1:1 ratio of beads to sample, followed by quantification on a Qubit 4 using the 1X dsDNA HS Assay Kit (Life Technologies). Quality control was performed using a High-Sensitivity DNA chip on a 2100 Bioanalyzer (Agilent Technologies, Santa Clara, CA, USA). Then, 10 ng of DNA from each post-Nextera library was pooled and sequenced on an Illumina NextSeq 550^®^ instrument using a 1 × 75 cycles high-output kit at the Bioinformatics and Expression Analysis Core facility, Karolinska Institutet, Stockholm.

### 4.6. RNA-Sequencing Data Processing and Analysis

A quality check of raw sequencing reads was performed with FastQC and MultiQC [26]. RNA sequencing (RNA-seq) data analysis was performed with Partek Flow Genomic Analysis Software (Build version11.0.24.0624 & database Version 508) (Partek Inc., St. Louis, MO, USA). Briefly, the FASTQ files were processed to filter the contaminants, such as ribosomal DNA and mitochondrial DNA, using a Bowtie 2 aligner, and we then trimmed the standard Nextera Transposase adapter (CTGTCTCTTATACACATCT) from the raw reads. The filtered reads were then aligned to the Hg38 genome using a STAR aligner with default settings. The total alignment rate was in the range of 95–99%—the unique alignment rate was 80–92%, with an average Phred quality score of 34 per base post-alignment. The filtered alignments were quantified for hg38 Ensembl Transcripts release 100. The obtained gene features were filtered where the value was less than 1 count in at least 80% of samples, and a total of 26,581 genes (78%) passed the criteria. Differential expression analysis was performed using the DEseq2 tool [27] on the Partek platform. The raw data and processed data files were submitted to Gene Expression Omnibus database with the GEO accession ID GSE252145.

### 4.7. Gene Ontology and Pathway Analysis

Gene ontology (GO) analyses for the functional annotation of the DEGs and enriched pathway analysis were conducted using the g:Profiler database (version e101_eg48_p14_baf17f0) with the Benjamini–Hochberg FDR multiple testing correction method, applying a significance threshold of 0.05 [28]. Reactome pathway analysis [29] and the Metascape-designet database [30] were also used.

### 4.8. RT-PCR Analysis

The extracted RNA samples were converted to cDNA using a SuperScript^®^ VILOTM kit (Invitrogen^®^, Thermo Fisher Scientific, Waltham, MA, USA). We technically validated a few significantly altered genes obtained by sequencing using Taqman^®^ gene probes, namely *CCL18* (assay ID: Hs00268113_m1), *MMP2* (assay ID: Hs01548727_m1), *COL1A1* (assay ID: Hs00164004_m1), *COL1A2* (assay ID: Hs01028956_m1), *COL3A1* (assay ID: Hs00943809_m1), and *18s* (4319413E) as a housekeeping gene (Thermo Fisher Scientific, Walthem, MA, USA). We designed a customized primer sequence for *ADAMTS2-1* (Forward primer sequence ‘cctgacaacccctacttttgc’; reverse primer sequence ‘tgaggatgtcaggtgtcagc’) and performed RT-PCR using a Sybr-green PCR assay. Then, 20 ng of cDNA from 10 samples was used in triplicate in the RT-PCR and analyzed on a One Step Plus Real-time PCR system (Applied Biosystems, Foster City, CA, USA), according to the manufacturer protocol. Fold change was calculated using the comparative Ct-method. A paired *t*-test compared the pre- and post-mifepristone treatment groups. To assess the impact of different doses of mifepristone on breast cells in vitro, a two-way ANOVA test was applied after the square-root transformation. Significance was considered at a *p*-value < 0.05. GraphPad Prism 9.1.2 (GraphPad Software Inc., San Diego, CA, USA) was utilized for the statistical analyses.

### 4.9. In Vitro Validation via Primary Epithelial Cell Isolation

After breast tissue collection and examination by a pathologist, the sample was transferred for tissue digestion and single-cell isolation. In brief, the tissue was washed in HBSS and manually diced on ice using DMEM with 10 mM HEPES and 0.1% (*w*/*v*) BSA. The small tissue fragments were transferred to a mixture of digestion enzymes (hyaluronidase and collagenase 1; Stemcell Technologies Catalog # 07912) in Epicult-C and incubated on a rotator at 37 °C for 4–18 h. The digested tissue was then filtered through a 100 μM strainer, and the resulting flowthrough was cultured (37°/5%) using a cocktail of serum-free Epicult-C (Stemcell Technologies Catalog # 05630) and Mammary Epithelial Cell Growth Medium (PromoCell C-21010).

### 4.10. Immunofluorescence

Following cell isolation, a total of 50,000 cells were seeded per well in eight-well NuncLab-Tek Chamber Slides (Sigma, St. Louis, MO, USA) and incubated for 72 h at 37 °C in a 5% CO_2_ incubator. Subsequently, the cells were fixed with 4% paraformaldehyde for 15 min at room temperature and then blocked with 2% BSA and 0.1% Triton-X (Sigma) in PBS for 30 min at room temperature. Subsequently, the cells were incubated with primary antibodies (CD49f, Rat, #MA5-16884, ThermoFisher Scientific (Waltham, MA, USA); EPCAM, Goat, R and D Systems Cat# AF960, RRID:AB 355745; CK8, Mouse, Santacruz, sc-8020; CK14, Rabbit, Invitrogen, # MA5-32214) in 2% BSA in PBS for 1 h at RT. After three washes with PBS, the cells were incubated with the respective secondary antibodies (Alexa Fluor; Donkey anti-Rat 488, Donkey anti-Goat 594, Donkey anti-Mouse 488, and Goat anti-Rabbit 594) in 2% BSA in PBS for 30 min at RT. Finally, the samples were mounted using ProLong Gold Antifade Mounting Medium with DAPI (imaging: Confocal Microscope Zeiss LSM700).

### 4.11. In Vitro Drug Treatment Assay

Primary isolated breast epithelial cells were cultured at a density of 5 × 104 cells per well in a 12-well plate. On the following day, these cells were subjected to different concentrations of mifepristone treatment (0, 5, 50, and 100 μM) for a duration of three days. Subsequently, the cells were collected, and their lysates were prepared at three distinct time points: the baseline before the initiation of treatment, after one day of treatment, and upon the completion of the three-day treatment period. To ensure robustness and reliability, we employed cells from three different donors for this study. For each experiment, we conducted three independent replicates.

### 4.12. In Silico Data Analysis

We employed the R2 Genomics Analysis and Visualization Platform [31] for conducting comparative transcriptomic analysis. This online resource facilitated the examination and assessment of the enrichment gene signature within the breast cancer cohort obtained from TCGA. The user-friendly interface and comprehensive tools provided by R2 allowed for efficiently exploring and statistically validating each gene within the enriched gene cohort. Our approach involved evaluating the differences between normal breast tissue and breast cancer, along with an exploration of their correlation with PR expression and signaling. To ensure statistical robustness, we applied a *t*-test with false discovery rate correction as a multiple testing approach, employing a *p*-value cutoff of 0.05.

## 5. Conclusions

In conclusion, our investigation into PR modulation in normal breast tissue, following mifepristone treatment, uncovers crucial alterations in gene expression patterns. The observed shifts in gene expression in pathways related to ECM organization point to the complicated involvement of ECM dynamics. The substantial linkage of our enriched signature with the PR expression in breast cancer emphasizes the downstream impact of progesterone. Undoubtedly, based on these descriptive data, comprehensive studies specifically designed to delve into the detailed molecular landscape alterations induced by mifepristone treatment could shed light on the molecular actions of progesterone. Furthermore, such studies may explore whether antagonizing progesterone could exhibit protective properties in the breast.

## Figures and Tables

**Table 1:** Upregulated and downregulated differentially expressed genes (DEGs) at baseline and after treatment with mifepristone.

Gene Name	*p*-Value	FDR	Fold Change
**A. Upregulated genes**			
CCL18	2.41 × 10^−6^	0.0041	16.0
WNT2	3.40 × 10^−6^	0.0047	5.1
CTSG	7.82 × 10^−5^	0.0397	4.5
TPSB2	5.50 × 10^−10^	0.0001	4.0
TPSAB1	6.58 × 10^−8^	0.0003	4.0
PIEZO2	1.54 × 10^−5^	0.0132	3.7
COL1A1	3.48 × 10^−6^	0.0047	3.6
DPP4	2.17 × 10^−8^	0.0002	3.6
GRIA3	3.44 × 10^−6^	0.0047	3.2
C1QTNF3	8.00 × 10^−5^	0.0397	3.0
COL1A2	2.69 × 10^−5^	0.0196	2.6
COL3A1	2.70 × 10^−5^	0.0196	2.6
OSR2	7.28 × 10^−8^	0.0003	2.4
CPZ	1.30 × 10^−6^	0.0027	2.3
ADAMTS2	2.96 × 10^−5^	0.0206	2.3
COL5A1	5.93 × 10^−7^	0.0019	2.2
MMP2	3.90 × 10^−5^	0.0245	2.1
GXYLT2	1.10 × 10^−4^	0.0480	2.1
ABI3BP	1.06 × 10^−4^	0.0477	2.0
**B. Downregulated genes**			
ZNF620	6.26 × 10^−5^	0.03	−2.16
LAMA1	3.59 × 10^−5^	0.02	−2.34
PRR4	8.47 × 10^−5^	0.04	−2.95
ASPRV1	5.73 × 10^−5^	0.03	−3.64
SLC4A11	1.38 × 10^−5^	0.01	−5.68
CCDC157	1.83 × 10^−5^	0.02	−5.96
IL1B	8.56 × 10^−5^	0.04	−6.06
RP1	7.80 × 10^−6^	0.01	−13.00
FDR = false discovery rate			

**Table 2:** Top 15 enriched gene ontology terms of differentially expressed upregulated genes, as determined by gene ontology (GO) analyses using g:Profiler database.

Term ID	Description	FDR (padj)
**Biological Process**		
GO:0030198	Extracellular matrix organization	2.21 × 10^−8^
GO:0043062	Extracellular structure organization	2.21 × 10^−8^
GO:0030199	Collagen fibril organization	6.95 × 10^−7^
GO:0032963	Collagen metabolic process	1.76541 × 10^−5^
GO:0071230	Cellular response to amino acid stimulus	0.000123191
**Cellular Component**		
GO:0062023	Collagen-containing extracellular matrix	4.78 × 10^−12^
GO:0031012	Extracellular matrix	4.59 × 10^−11^
GO:0098643	Banded collagen fibril	1.13 × 10^−8^
GO:0005583	Fibrillar collagen trimer	1.13 × 10^−8^
GO:0098644	Complex of collagen trimers	1.08 × 10^−7^
**Molecular Function**		
GO:0048407	Platelet-derived growth factor binding	2.76 × 10^−8^
GO:0030020	Extracellular matrix structural constituent conferring tensile strength	4.1495 × 10^−6^
GO:0005201	Extracellular matrix structural constituent	1.75112 × 10^−5^
GO:0004252	Serine-type endopeptidase activity	1.75112 × 10^−5^
GO:0017171	Serine hydrolase activity	2.17779 × 10^−5^
FDR = false discovery rate		

## Data Availability

The raw data and processed data files were submitted to Gene Expression Omnibus database with the GEO accession ID GSE252145 (for reviewer access, please go to https://www.ncbi.nlm.nih.gov/geo/query/acc.cgi?acc=GSE252145 and enter token qfqnyscmvzmzjcp into the box). (Accessed on 27 December 2023).

## Supplementary Materials

The following supporting information can be downloaded at: https://www.mdpi.com/article/10.3390/ijms25147590/s1.
