# Supplementary material for: Transcriptomic Profile of Breast Tissue of Premenopausal Women Following Treatment with Progesterone Receptor Modulator: Secondary Outcomes of a Randomized Controlled Trial
Source: Int J Mol Sci. 2024 Jul 10;25(14):7590. doi: 10.3390/ijms25147590 (PMC11277027; doi:10.3390/ijms25147590)
Supplement: Supplementary file 1 [file ijms-25-07590-s001.zip › ijms-3055523-supplementary.pdf]

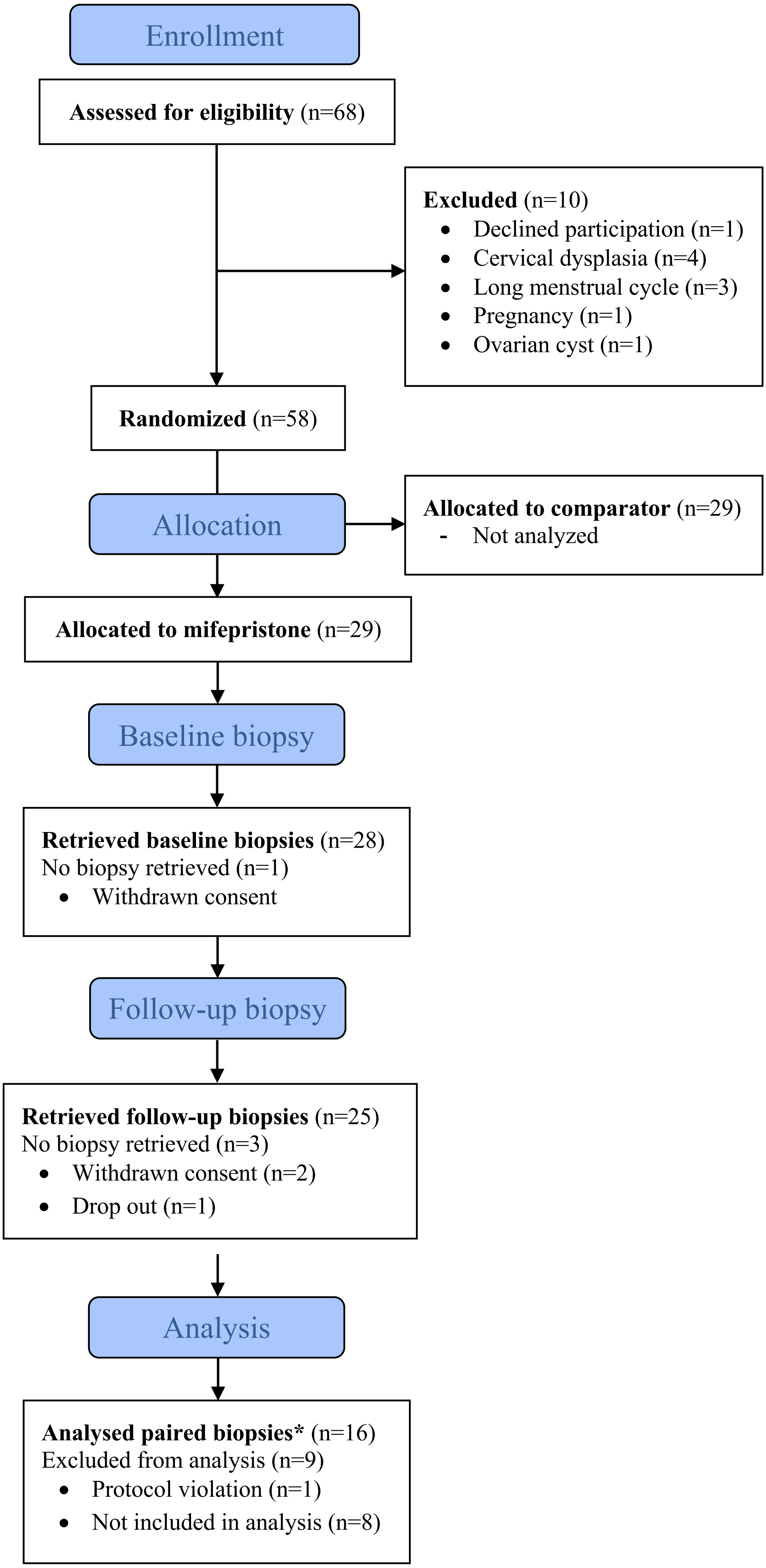
Enrollment
Assessed for eligibility (n=68)
Excluded (n=10)
Declined participation (n=1)
Cervical dysplasia (n=4)
Long menstrual cycle (n=3)
Pregnancy (n=1)
Ovarian cyst (n=1)
Randomized (n=58)
Allocation
Allocated to comparator (n=29)
- Not analyzed
Allocated to mifepristone (n=29)
Baseline biopsy
Retrieved baseline biopsies (n=28)
No biopsy retrieved (n=1)
Withdrawn consent
Follow-up biopsy
Retrieved follow-up biopsies (n=25)
No biopsy retrieved (n=3)
Withdrawn consent (n=2)
Drop out (n=1)
Analysis
Analysed paired biopsies* (n=16)
Excluded from analysis (n=9)
Protocol violation (n=1)
Not included in analysis (n=8)

Figure S1

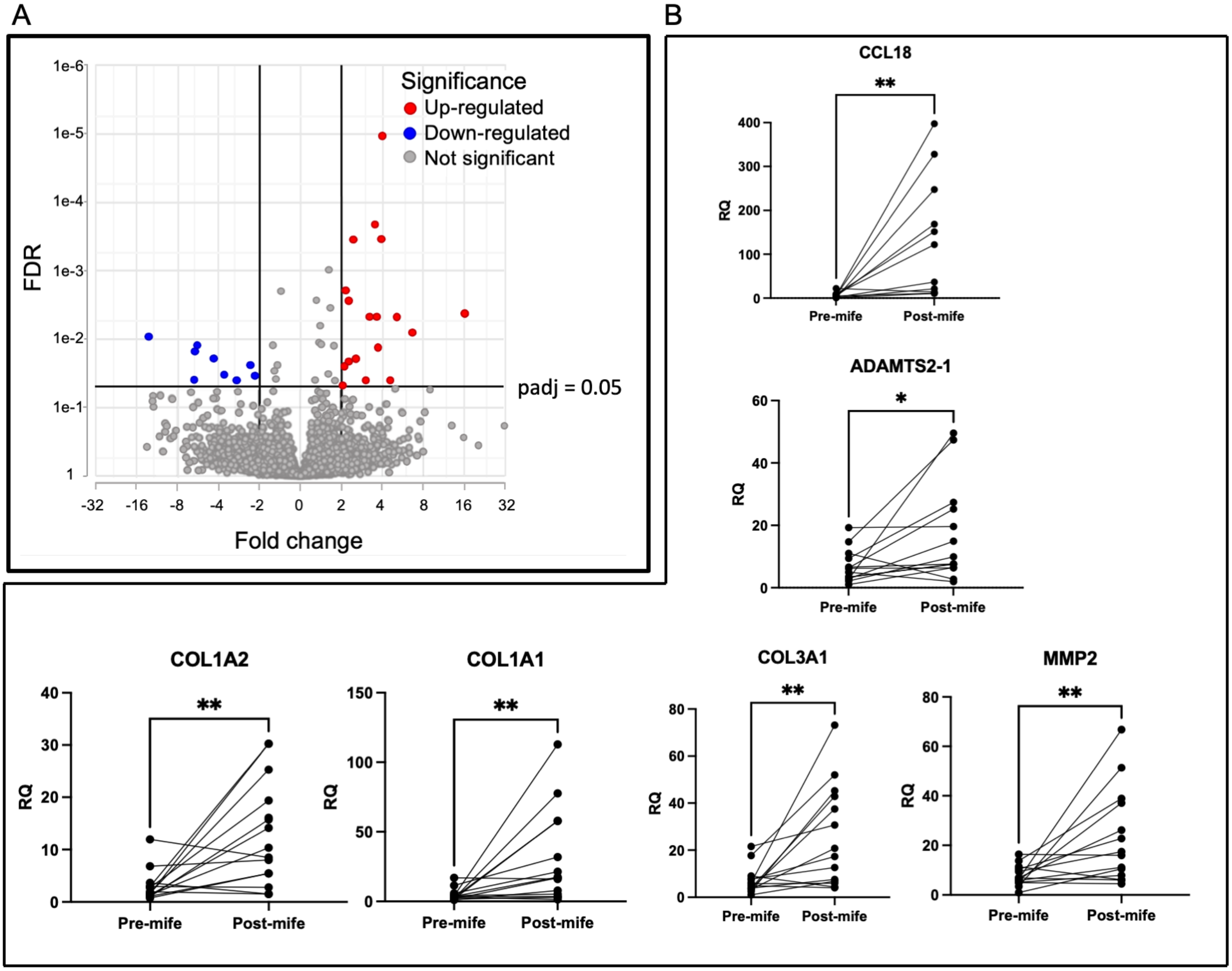
A
B
FDR
Fold change
Significance
Up-regulated
Down-regulated
Not significant
padj = 0.05
1e-6
1e-5
1e-4
1e-3
1e-2
1e-1
1
-32
-16
-8
-4
-2
0
2
4
8
16
32
CCL18
ADAMTS2-1
COL1A2
COL1A1
COL3A1
MMP2
RQ
Pre-mife
Post-mife
**
*

Figure S2

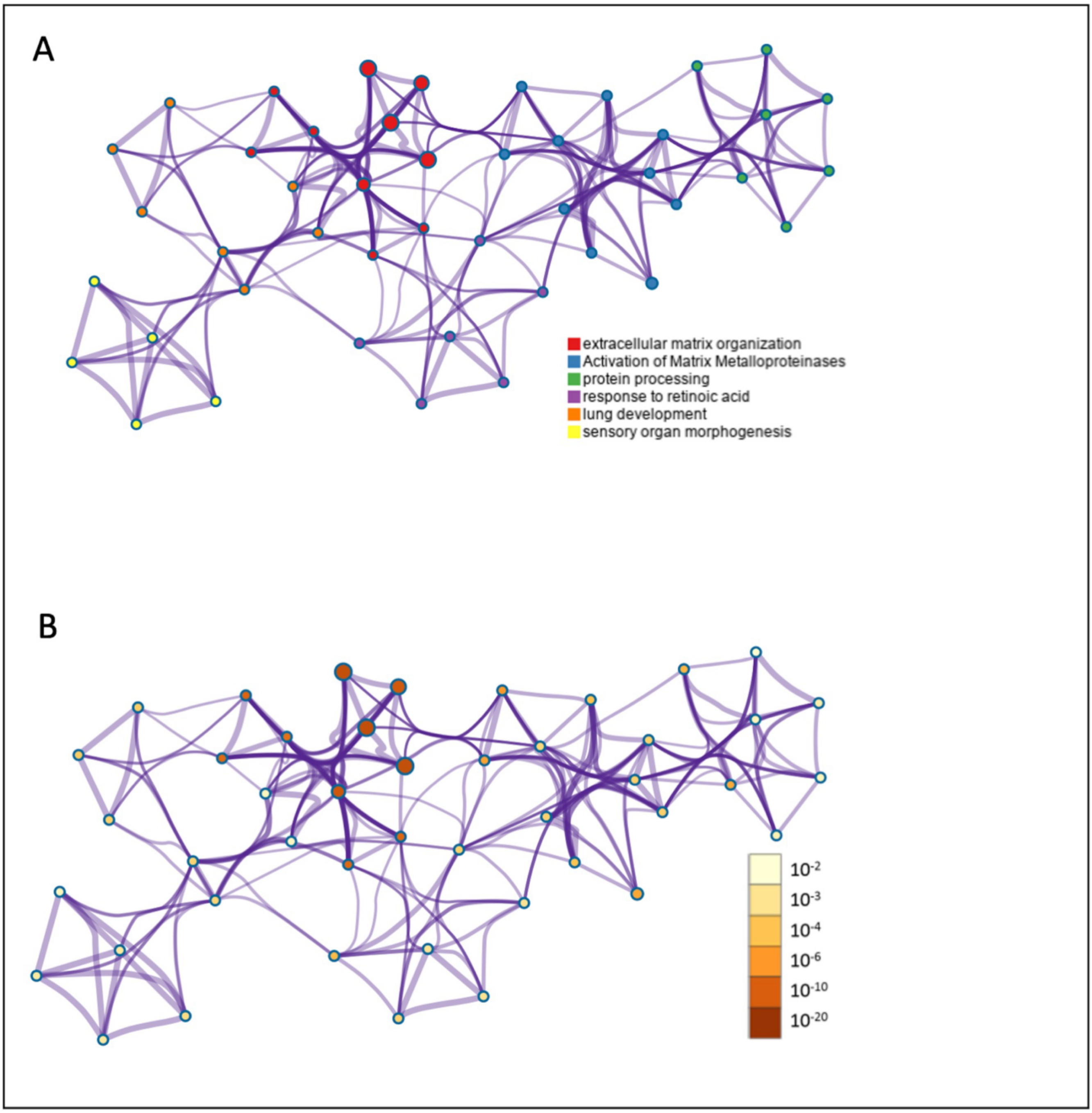
A
extracellular matrix organization
Activation of Matrix Metalloproteinases
protein processing
response to retinoic acid
lung development
sensory organ morphogenesis
B
10-2
10-3
10-4
10-6
10-10
10-20

Figure S3

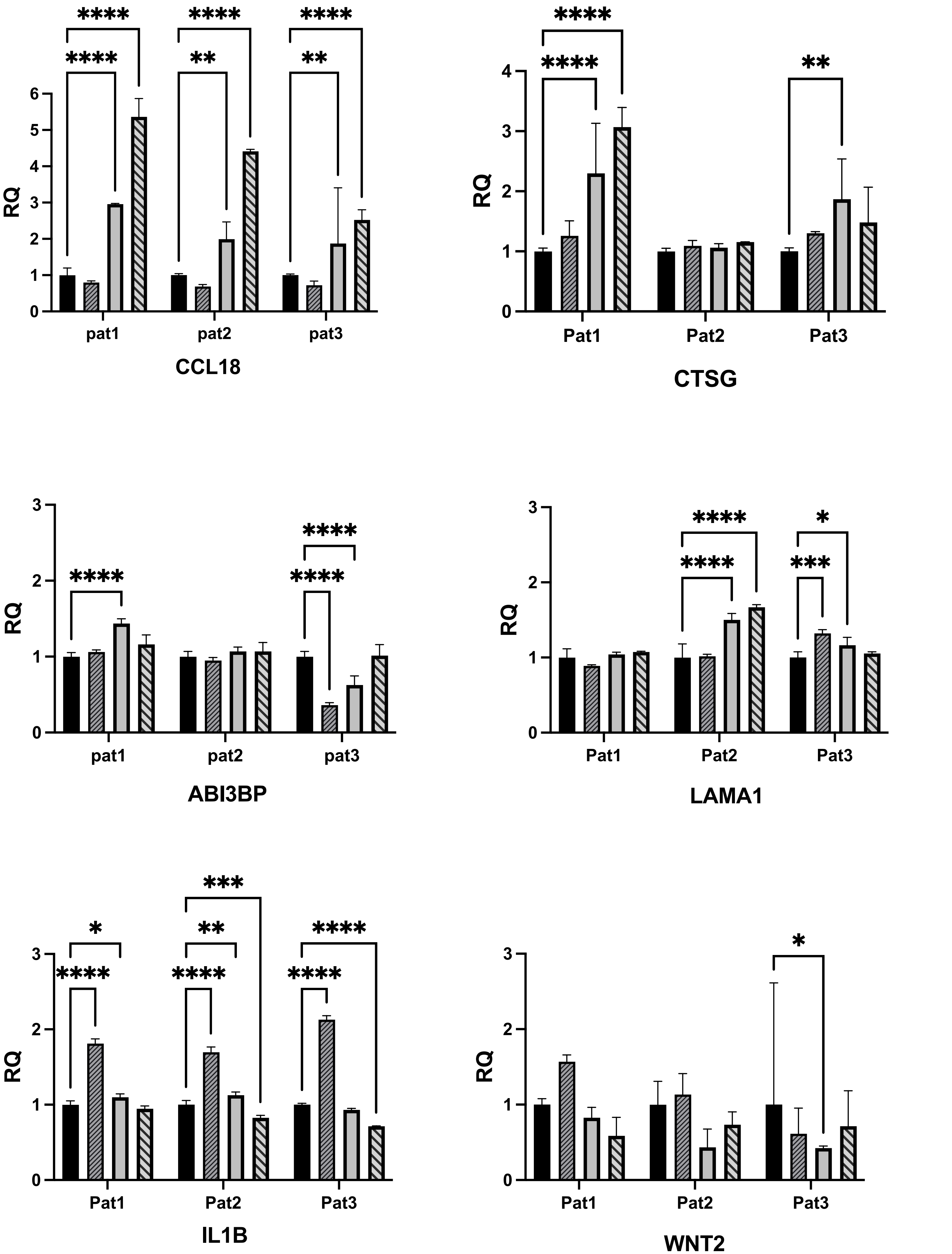
CCL18
CTSG
ABI3BP
LAMA1
IL1B
WNT2
RQ
pat1
pat2
pat3
Pat1
Pat2
Pat3

Figure S4

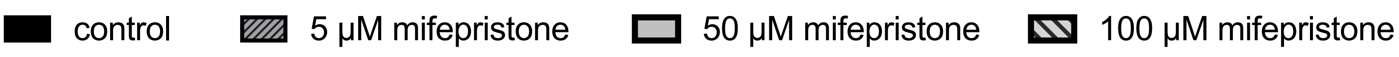
control
5 μM mifepristone
50 μM mifepristone
100 μM mifepristone

Supplemental table S1. Baseline characteristics of women contributing to paired breast biopsies, before and after mifepristone treatment, expressed as median (range).

Age (years)	33 (21 – 41)
Pregnancies	1.5 (0 – 5)
Parity	2 (0 – 3)
Cycle length (days)	29 (27 – 32)
Menstrual period (days)	5 (3 – 7)
Body Mass Index	25.8 (18.4 – 32.0)

Supplemental table S2. Top 10 enriched pathways of the upregulated differently expressed genes as determined by Reactome pathway analysis.

Pathway ID	Name	FDR (Padj)	Genes
R-HSA-1474228	Degradation of the extracellular matrix	1.80E-09	CTSG. TPSAB1. COL1A1. COL1A2. COL3A1. COL5A1. MMP2
R-HSA-1474244	Extracellular matrix organization	2.57E-09	CTSG. TPSAB1. COL1A1. COL1A2. COL3A1. ADAMTS2. COL5A1. MMP2
R-HSA-1650814	Collagen biosynthesis and modifying enzymes	9.22E-08	COL1A1. COL1A2. COL3A1. ADAMTS2. COL5A1
R-HSA-1442490	Collagen degradation	9.22E-08	COL1A1. COL1A2. COL3A1. COL5A1. MMP2
R-HSA-3000170	Syndecan interactions	1.76E-07	COL1A1. COL1A2. COL3A1. COL5A1
R-HSA-1474290	Collagen formation	2.30E-07	COL1A1. COL1A2. COL3A1. ADAMTS2. COL5A1
R-HSA-8874081	MET activates PTK2 signaling	2.30E-07	COL1A1. COL1A2. COL3A1. COL5A1
R-HSA-8875878	MET promotes cell motility	6.69E-07	COL1A1. COL1A2. COL3A1. COL5A1
R-HSA-8948216	Collagen chain trimerization	8.81E-07	COL1A1. COL1A2. COL3A1. COL5A1
R-HSA-3000171	Non-integrin membrane-ECM interactions	2.460x10-6	COL1A1. COL1A2. COL3A1. COL5A1

FDR=false discovery rate
